# The Monkeypox Outbreak and Implications for Dental Practice

**DOI:** 10.1016/j.identj.2022.07.006

**Published:** 2022-08-05

**Authors:** Lakshman Samaranayake, Sukumaran Anil

**Affiliations:** a Faculty of Dentistry, The University of Hong Kong, Hong Kong Special Administrative Region, China; b Department of Dentistry, Oral Health Institute, Hamad Medical Corporation, Doha, Qatar; c College of Dental Medicine, Qatar University, Doha, Qatar

**Keywords:** Monkeypox, MPX virus, Dentistry, Infection control, Oral manifestations

## Abstract

**Objectives:**

Monkeypox (MPX) caused by the MPX virus, is a contagious disease confined mainly to African regions, and is currently making multiple appearances outside of disease-endemic countries. World Health Organization (WHO) very recently declared the current monkeypox outbreak a Public Health Emergency of International Concern. We review here the salient features of MPX and its possible impact on dentistry.

**Methods:**

The data on the aetiology, transmission modes, signs and symptoms, diagnosis, and management, including the risk of its occupational transmission in dental settings, were garnered from the current literature, mainly from the World Health Organization and Centers for Disease Control and Prevention databases.

**Results:**

Over recent months, MPX has reemerged in more than 88 countries in Europe, North America, and Australia, with some 22000 case reports to date (as of July 2022). The initial signs of MPX appear during the prodromal period, in the oral cavity as single or multiple macular lesions on the oral mucosa, accompanied by generalised lymphadenopathy. Subsequently, the characteristic rash appears on the skin and spreads centripetally from the trunk towards the palms and soles. MPX is a self-limiting disease with very low mortality and may last from 2 to 4 weeks. Although MPX is similar to chickenpox, there are a number of differentiating signs, the main element being lymphadenopathy. Strict adherence to standard, contact, and droplet infection control precautions, including wearing N95 masks, FFP3 respirators, fluid-resistant attire, and eye protection, is necessary to prevent its spread.

**Conclusions:**

MPX appears to be a significant travel-related disease. Dental care workers should note that premonitory signs of the disease usually appear on the oral mucosa as macules and ulcers prior to the characteristic skin lesions. Implementing standard, contact, and droplet infection control measures, patient isolation, and referral are important, particularly during a local outbreak. A vaccine specific for MPX is under development, although the smallpox vaccine appears to be effective.

## Introduction

Monkeypox (MPX), caused by the monkeypox virus (MPXV), is a highly contagious infectious disease first documented in humans in the 1970s. Sporadic outbreaks of MPX have been reported in many countries, though most cases have been restricted to endemic areas in the African continent. However, the rapid emergence of MPX in a number of cities outside the virus's endemic region amongst individuals who are not necessarily interconnected or who have not traveled to Africa, along with the recent evidence indicating person-to-person transmission, has been a major concern for many health authorities.^1^

Most cases reported in nonendemic countries are amongst gay, bisexual, and other men who had sex with men. In contrast to the previous sporadic cases, the current outbreak has affected human communities with no travel links to an endemic African region.^2^ At the time of this writing (30 July 2022), more than 22000 cases in some 88 countries have been confirmed in Europe, North America, and Australia.^3^

Several hypotheses have been presented to explain the current outbreak.^4^ The most favoured is the general decline in the population immunity to smallpox and similar *Orthopoxvirus* diseases, as the smallpox vaccination programme was discontinued 30 years ago. As mentioned, during the current outbreak, a high incidence was recorded amongst adults aged 21 to 40 who were unvaccinated against smallpox.^5^ In particular, viral transmission appears to be common amongst men who engage in sex with other men with prolonged skin-to-skin contact.^2^^,^^6^

In endemic settings, MPXV enters communities through zoonotic transmission instead of sustained human-to-human transmission.^4^^,^^7^ It is believed that small mammals, such as elephant shrews and rodents, play a role in the natural history of the virus, although the reservoir host remains elusive as yet.^8^ MPX is usually a self-limiting disease with symptoms lasting between 2 and 4 weeks.

In general, the case fatality rates of the current epidemic have been between 3% and 6% and are likely to be higher amongst children, although the reasons for this are yet unknown.^3^^,^^9^ However, the child mortality rates appear to be proportional to the amount of virus exposure and the patient's general health status.^4^^,^^10^

This concise review provides data on the current outbreak of MPX and discusses its modes of transmission, signs and symptoms, diagnosis and management, and its occupational transmission risk in dental settings.

## Virology

**Fig. 1:**
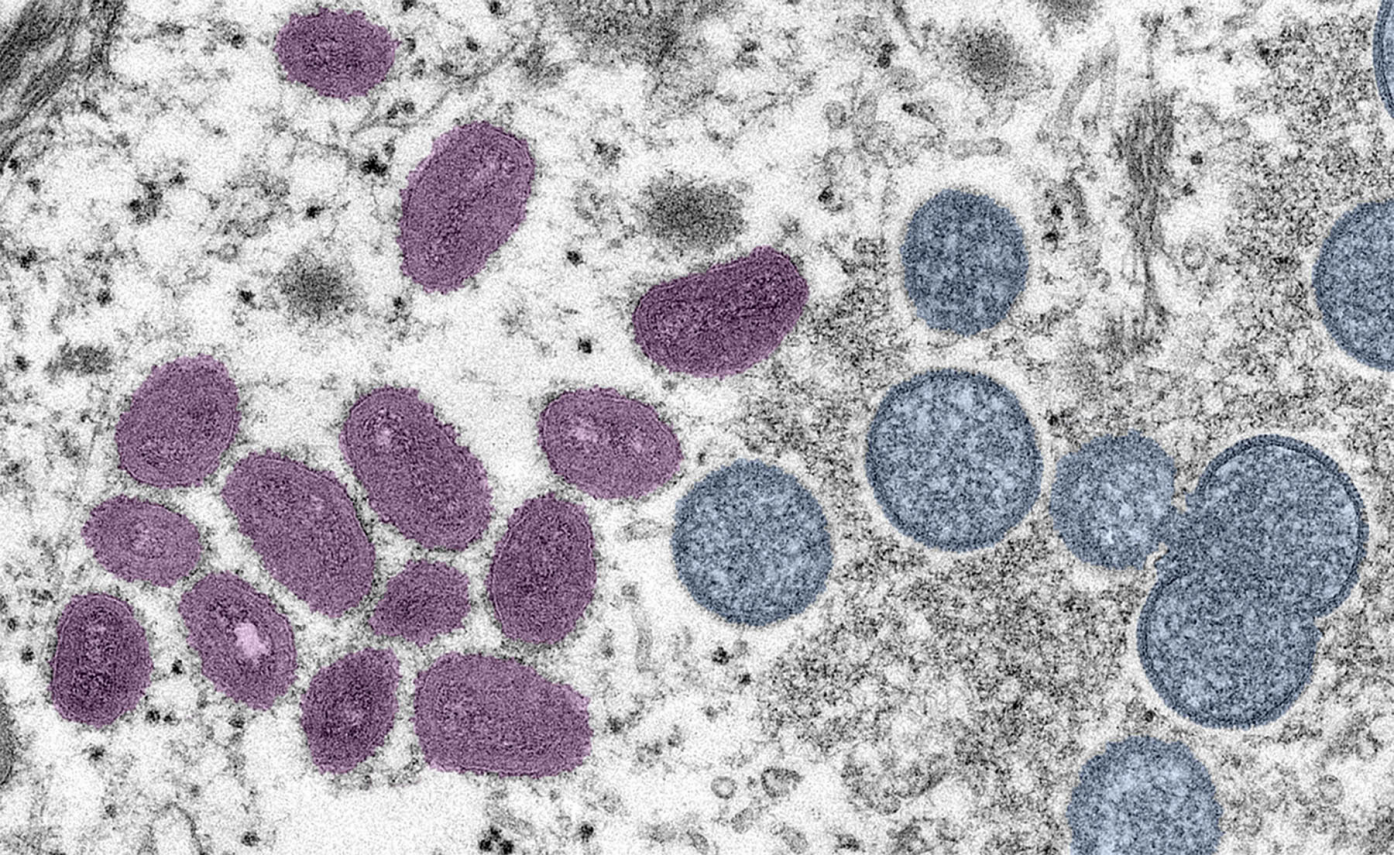
A negative stain, pseudo colour, electron micrograph showing mature, oval, mulberry-shaped virus particles (pink) and the immature particles (blue) from a skin lesion of a patient with monkeypox. (Photo credit: Cynthia S. Goldsmith, Russell Regnery; Courtesy CDC Image library.)

MPXV is a double-stranded DNA virus that belongs to the Poxviridae family, Chordopoxvirinae subfamily, and *Orthopoxvirus* genus. It has a characteristic ultrastructure with mature mulberry shaped viral particles (Figure 1). Although MPXV has a low mutation rate, under certain selective pressure, adaptive mutations of the MPXV may occur, and that might have enhanced its transmissibility. Additionally, recent studies indicate that the MPXVs in this outbreak exhibit single nucleotide alterations and frameshift mutations compared to those in previous episodes.^11^

The West African (Congo Basin) and Central African clades of this virus, named after their geographic distribution, cause a similar clinical condition but with varying severity.^8^^,^^12^ In the Congo Basin clade, death rates ranging from 1% to 10% have been documented, and the viral lineage circulating in this area appears to be associated with higher virulence.^13^

The West African clade is linked to a generally lower fatality rate, typically less than 3%.^14^ Significant variations exist in mortality rates, which are subject to case ascertainment bias. Pneumonitis, encephalitis, sight-threatening keratitis, and subsequent bacterial infections are some of the severe side effects of MPX.^15^ The MPXV, which belongs to the Central African clade, is more susceptible to human-to-human transmission because it is more virulent than in the West African clade.^16^ Cameroon, the only country where both virus clades have caused infections, serves as the geographic divide between the 2 virus clades.

Experimentally, monkeys have developed MPX after intradermal, subcutaneous, intramuscular, and intravenous inoculation with MPXV. Clinical and subclinical infections develop in unvaccinated companion monkeys confined in separate cages with experimentally infected animals.^12^ The likely transmission mode for the latter infections was considered to be the airborne, respiratory route, whilst autoinoculation or ingestion of viral particles could not be ruled out.^17^

Although there are many animal pox diseases such as cowpox, sheeppox, and fowlpox, only 3 major human pox infections are worthy of concern. These are smallpox, chickenpox, and MPX.^18^ Of these, smallpox has now been eradicated, and the main contemporaneous clinical diagnostic issue is the differentiation between human MPX and chickenpox. The presence of lymphadenopathy, prodromal high fever, and slower maturation of skin lesions and dark scab formation are the critical differentiating signs supporting a correct diagnosis of MPX^19^ (Table).

**Table:** Differentiating features of MPX and chickenpox.

	MPX	Chickenpox
Agent	MPX virus *Orthopoxvirus* family	Varicella zoster virus
Spread (similar)	Close contact, respiratory droplets, contact with skin lesions, recently contaminated objects	
Contagiousness	High	Relatively low
Incubation period	Averages 7−14 days but can range from 5−21 days	10−21 days
Illness	Mild illness with distinctive swollen lymph nodes (lymphadenopathy); initial symptoms include fever, headache, muscle aches, backache, chills, and exhaustion	Swollen lymph nodes appear rarely if ever; other symptoms are fever, tiredness, headache, and loss of appetite
Symptoms	Lasts 2−4 weeks	Lasts up to 2 weeks; commonly subsides in 1 week
Fever	1−5 days before rash	1−2 days before rash
Rash characteristics	Generally, begins on the face then spreads to other parts of the body, including palms and soles; the rash eventually forms a scab that falls off	Itchy, blister-like rash, first on the chest, back, and face and then over the entire body; absent on palms and soles and unlikely to form scabs
Oral lesions	Affects the oral mucosa in 70% of cases as macules and vesicles; lips may also be affected	May appear in the mouth before skin lesions, on either the keratinised or nonkeratinised mucosa
Fatality	Some strains cause severe disease, with fatality in 3% to 6% (WHO)	Rare; any seen are typically in patients with comorbidities
Isolation	3 weeks; avoid contact with immunosuppressed people, pregnant women, and children younger than 12 years	From 1–2 days before rash onset until lesions are crusted (generally 4–6 days after rash onset)
Prevention	The traditional smallpox vaccine is protective against MPX; a vaccine approved for the prevention of MPX is also available, based on a strain of vaccinia virus (known generically as modified vaccinia Ankara Bavarian Nordic strain, or MVA-BN)	Chickenpox or varicella vaccine (2 doses 4 to 6 weeks apart) contains attenuated live varicella zoster virus; a combination vaccine of measles, mumps, rubella, and varicella (MMRV) is now available; varicella zoster immunoglobulin (VZIG) may be given for those exposed to infectious individuals
Vaccine recommendation for HCWs	Desirable for those in MPX-endemic regions or in the case of an outbreak	Recommended by WHO for HCWs who are not immune due to natural infection

Sources: Various, including World Health Organization (WHO) and US Centers for Disease Control and Prevention (CDC).

HCW, health care worker; MPX, monkeypox.

## Epidemiology

MPX is a zoonotic virus, implying that it is spread from animals to humans through close contact, such as a bite, scratch, or contact with material from MPX lesions and through large respiratory droplets.^20^

### Human-to-human transmission

Human-to-human transmission of MPX has been uncommon in the past. Direct contact with lesion material or respiratory droplets has been identified as the mechanism of person-to-person transmission. Previous workers also noted that sleeping in the same room or bed, living in the same household, and drinking or eating from the same dish increased the risk of contracting the virus through human-to-human transmission.^7^^,^^21^^,^^22^

The infection spreads through large respiratory droplets and typically requires prolonged close contact. This is in contrast to the current SARS-CoV-2 infection that can spread through even very small droplets.^23^ Experimental studies have shown that MPXV may remain infective in aerosols for several hours,^24^^,^^25^ and infection may spread via aerosolised MPXV in animal models.^26^ The virus can also spread via bodily fluids such as blood and semen, although the salivary route of transmission has not been documented as yet.^27^

A number of recent cases have primarily involved men who have sex with men.^28^ Apart from these cases, human-to-human transmission is uncommon, yet there have been reports of at least 2 serial transmission events.^29^^,^^30^ Finally, in this context, other transmission routes, such as mother-to-child transmission or nosocomial infection in hospital settings, have been rarely documented.^10^^,^^31^^,^^32^

### Animal-to-human transmission

**Fig. 2:**
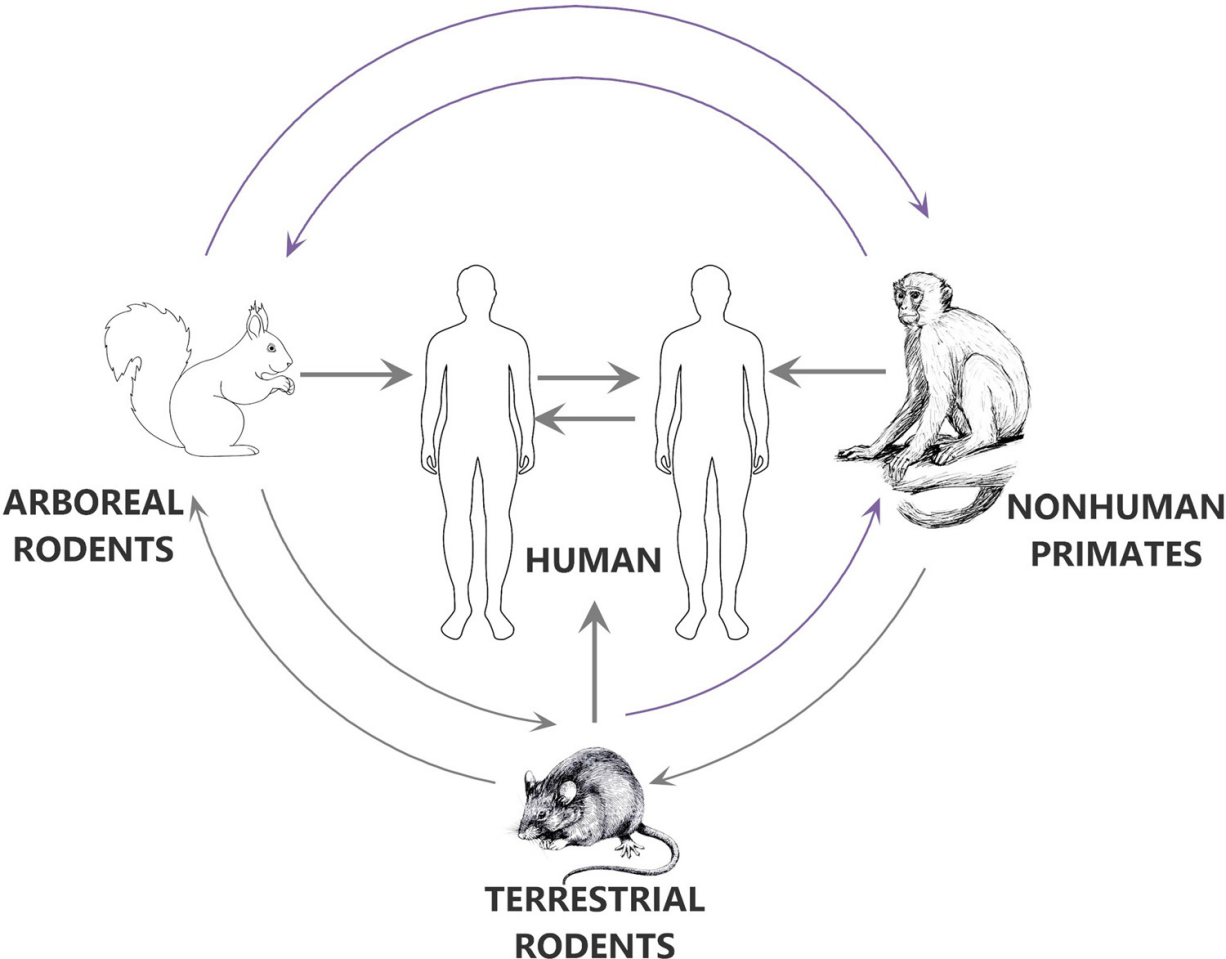
Modes of the spread of the monkeypox virus.

Animal-to-human transmission of MPXV may occur via animal bites or scratches by infected small mammals, including rodents and nonhuman primates, or through the consumption of bush meat (Figure 2). Additionally, direct contact with body fluids or lesions from an infected animal or contaminated material may lead to transmission events.^33^ Consuming undercooked meat from an infected animal is thought to be a potential additional risk factor.

Furthermore, in the tropics, sleeping outside or on the ground, living near the forest, or visiting the forest were identified as factors that increase the risk of exposure to animals and, consequently, the risk of MPX transmission from animals to humans.^34^^,^^35^ Despite these risk factors, animal-to-human transmission routes have not been documented in the current worldwide outbreak.

## Clinical features

### Incubation period

After entering through the oropharynx, nasopharynx, or skin, the MPXV replicates at the inoculation site and then spreads to lymph nodes. Following an initial viremia, the virus spreads to other organs. The incubation period typically lasts between 7 and 14 days and has a maximum duration of 21 days.^36^ Immune markers have demonstrated the presence of asymptomatic MPX infections in both smallpox-vaccinated and unvaccinated individuals.^37^

### Symptomatology

The early phase of clinical sickness typically lasts between 1 and 5 days, during which time patients may have a fever, headache, back pain, muscle aches, fatigue, and lymphadenopathy: a characteristic of this disease.^38^ This is followed by a second phase, which usually begins 1 to 3 days after the fever subsides and is characterised by the emergence of a rash.^39^ Over a period of 2 to 3 weeks, the rash progresses through various stages before crusting and desquamating. Lesions range in diameter from 0.5 to 1 cm and in quantity from a few to several thousand.^40^ In addition to the oral mucosa conjunctiva, cornea, and/or genitalia, the eruption is typically centrifugal, beginning on the face and spreading to the palms and soles of the hands and feet.^41^ Observations of current outbreaks in European and North American nations describe lesions that originate in the genital region.

**Fig. 3:**
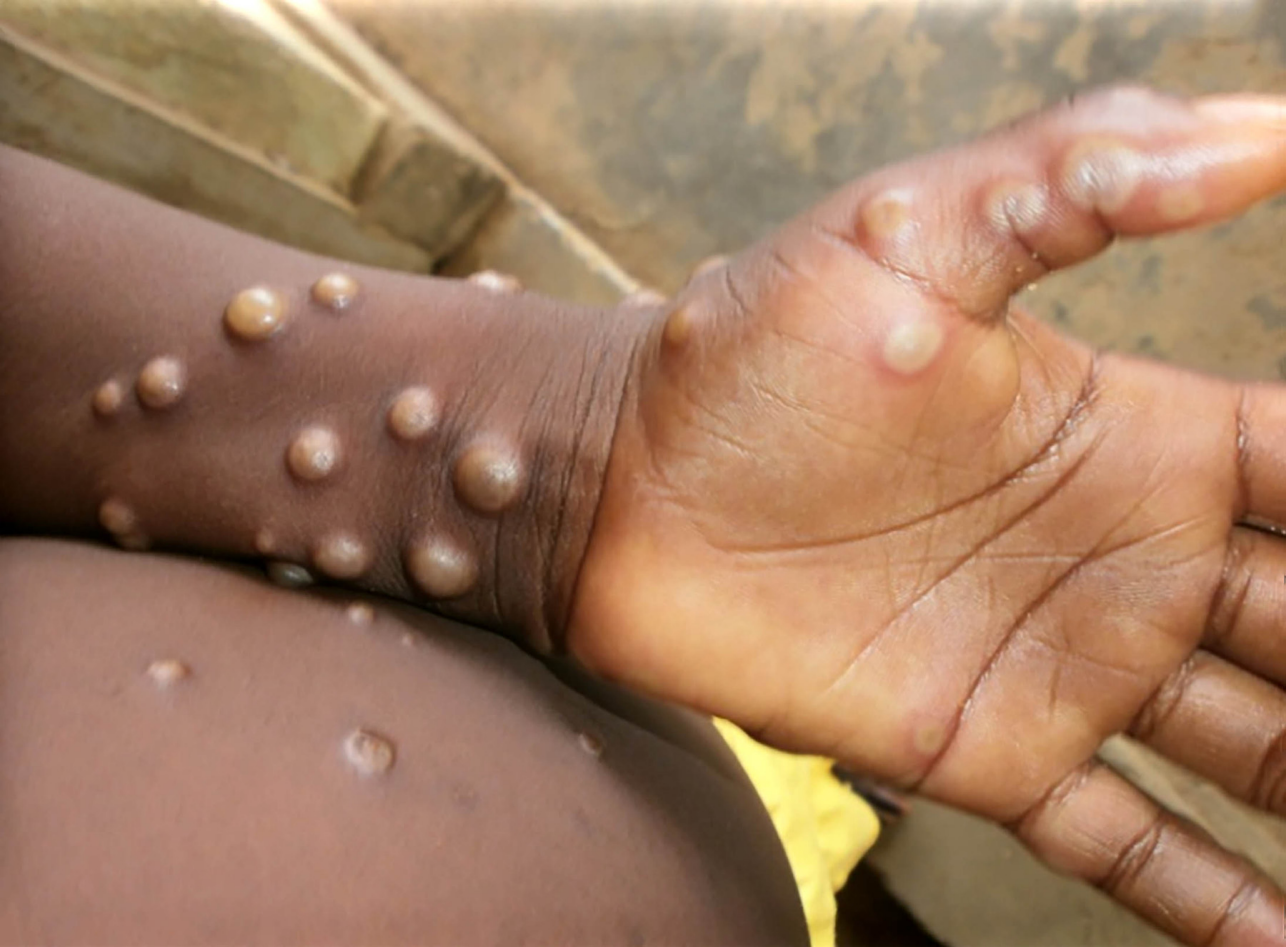
The wrist and palm of a patient with monkeypox, displaying the appearance of the papular and blister stage lesions prior to disruption and scab formation. (Photo credit: Nigeria Centre for Disease Control.)

The prodromal symptoms of MPX include fever, headache, myalgia, backache, chills, fatigue and, in particular, generalised lymphadenopathy, which distinguishes it from chickenpox (Table). Mucosal lesions in the mouth appear first, followed by skin lesions on the face and extremities, including palms and soles, and sometimes extending to the genital region. The rash will undergo the typical macular, papular, vesicular, and blister stages (Figure 3). Finally, the blisters break, forming a black scab that eventually falls off.^42^^,^^43^

Cases of MPX are typically mild, and patients usually recover within a few weeks. However, the mortality rate differs depending on the viral type. The European Centre for Disease Prevention and Control reports that the West African clade, observed in Europe, has a case fatality rate of approximately 3.6%. Children, young adults, and immunocompromised individuals have a higher mortality rate due to pneumonitis, encephalitis, sight-threatening keratitis, and subsequent bacterial infections.

Of interest in the dental context is that the primary lesions originate in the oropharynx before manifesting on the skin. Occasionally, oral ulcers can impair a patient's ability to eat and drink, causing dehydration and malnutrition.^44^ Perioral papules which blistered and ulcerated have been reported initially in the current outbreak.^27^ In one study, it was reported that oral ulcers were present in almost one-quarter (23.5%) of participants with MPX.^45^^,^^46^

Hence, in disease-endemic regions, dental care workers may be the first to detect the harbinger symptoms of MPX. Therefore, dental health care workers working in disease-prevalent or -endemic areas should maintain a high degree of suspicion, particularly when examining patients with lymphadenopathy. A thorough examination of the oral mucosa for macular, papular lesions in all patients is essential.

## Diagnosis

In light of the ongoing outbreak, clinicians evaluating patients with new-onset fever and rash should consider MPX, particularly if lymphadenopathy is also present. Typically, serum antibodies are detected around 2 weeks postexposure, when oral or skin lesions appear.^47^ A swab is used to collect crust or exudate from the lesion to isolate viral nucleic acids for diagnosis.^48^ The swabs and lesion aspirates demonstrate the strongest correlation with both infectiousness and the clinical course of infection.^1^ The western blot method, on the other hand, uses MPX virus proteins to verify its presence.^49^ The recommended test for identifying MPXV during an acute infection, as per the World Health Organization, is the real-time polymerase chain reaction test.^32^ In general, these tests are available at state laboratories for public health in the developed world. Commercial tests are not available at this point.

## Management

As MPX is a self-limiting infection, management is essentially symptomatic, and no specific antiviral agents are available. Nevertheless, the smallpox vaccine and the antiviral agents cidofovir, brincidofovir, and tecovirimat have activity against MPX and could be prescribed for patients with comorbidities.^50^ Passive immunisation with immune globulin vaccinia (6000 UI/kg to 24,000 UI/kg) has been successful.^51^

## Prevention

The disease can be contained by following the basic principles of infection control, which include rapid identification and isolation of the index patient, the use of personal protective equipment (PPE) by health care workers, and comprehensive contact tracing, which includes secondary case monitoring throughout the incubation period. Hospital systems should ensure that their health providers, particularly clinical staff, are aware of the infection control procedures, especially when dealing with infected patients. Any patient with a fever and disseminated vesicular or pustular rash should be immediately placed on airborne and contact precautions, as these are the classic symptoms of *Orthopoxvirus* infections.^40^

Vaccines against smallpox are effective in preventing MPX and postexposure prophylaxis. The US Food and Drug Administration has approved the JYNNEOS (Bavarian Nordic) smallpox vaccine to prevent MPX, and the ACAM2000 vaccine can be used off-label for the same purpose.^50^^,^^52^ Vaccination of close contacts has successfully limited transmission in previous outbreaks. If a prophylactic vaccine is administrated as early as possible after exposure, the infection can be prevented. Vaccinia immune globulin may be an alternative postexposure prophylactic agent when the smallpox vaccine is contraindicated.^49^

The Centers for Disease Control and Prevention also recognises the theoretical risk of airborne transmission and recommends implementing airborne infection control protocols whenever possible. These protocols include using N95 masks and other PPE when providing care or coming into close contact with an infected individual.

## Precautions in dental clinics

Clinicians must be alert for rashes resembling MPX and distinguish MPX from herpetic and similar vesicular-bullous lesions for differential diagnosis. Direct contact with MPX lesions or with the patient's belongings that have been in contact with lesions is the primary mode of transmission. Hence, in dental care settings, transmission can be prevented by taking standard, contact, and droplet infection control precautions when treating patients with symptoms of MPX.^18^^,^^53^^,^^54^ In addition, due to the potential risk of airborne transmission of MPXV, airborne precautions should be taken in accordance with the risk assessment, and N95 masks should be worn by all attending dental personnel. The patient should be treated in isolation, and precautions should be taken to minimise exposure to surrounding individuals, such as placing a surgical mask over the patient's nose and mouth and to cover any exposed skin lesions.

Principal preventative measures against infection with MPXV are provided in a document by the UK Health Security Agency and should be consulted. Some features of the recommendations are given below^55^:

• Elective dental treatment in patients with probable or confirmed MPX should be deferred until the patient is no longer infectious.
• If treatment is essential in acute cases, the patients should be treated in an isolated room rather than a shared treatment area.^55^
• Pregnant persons and severely immunocompromised individuals should not provide care for patients with MPX or those suspected of having an MPX infection.
• There should be strict adherence to standard, contact, and droplet infection control precautions in dentistry, including wearing N95 masks, FFP3 respirators, fluid-resistant attire, and eye protection.
• Perform proper hand hygiene after contact with infected individuals. For instance, hands should be washed with soap and water or cleaned by using a hand sanitiser containing alcohol.
• Avoid touching any materials that have come into contact with a patient with MPX.
• Cleaning and disinfecting should be strictly performed in the “dirty” areas of the dental facility, where patient care equipment is precleaned prior to sterilisation.
• Isolate infected patients particularly from those who may be susceptible to infection.
• Careful handling and collection of linens, hospital gowns, towels, and other fabrics is required.

## Conclusions

MPX has made multiple appearances outside of disease-endemic countries, indicating that it has become a significant travel-related disease, at least for the time being, and all health care workers including dental personnel should remain vigilant in preventing its spread. Indeed, the manner in which MPXV has simultaneously cropped up in several countries is rather mysterious. Currently, the virus is being sequenced in depth, to ascertain its origin as well as the likelihood of high transmissibility. The signs are that, in the longer term, it will be another short-lived sporadic viral infection of little or no major consequence to health professionals in the developing world, although dental personnel in regions in Africa where the disease is endemic should be persistently vigilant.

## Author contributions

LPS and SA conceived the idea for this article. LPS and SA wrote the manuscript, and LPS edited the manuscript. LPS and SA read and accepted the whole manuscript.

## Conflict of interest

None disclosed.
